# *Tuber elevatireticulatum* sp. nov., a new species of whitish truffle from Taiwan

**DOI:** 10.1186/s40529-018-0241-y

**Published:** 2018-10-29

**Authors:** Chieh-Lung Lin, Ming-Jer Tsai, Chuen-Hsu Fu, Tun-Tschu Chang, Hoi-Tung Li, King-Fai Wong

**Affiliations:** 1 Division of Watershed Management, Taiwan Forestry Research Institute, COA, Taipei, Taiwan; 2 Department of Forestry and Resource Conservation, National Taiwan University, Taipei, Taiwan; 3 The Experimental Forest, National Taiwan University, Nantou County, Taiwan; 4 Division of Forest Protection, Taiwan Forestry Research Institute, COA, Taipei, Taiwan; 5 Tianroei Limited Company, Taipei, Taiwan; 6 Advance Plant Protection Limited Company, Hsinchu, Taiwan

**Keywords:** *Keteleeria*, Morphology, Phylogeny, Taxonomy, Taiwan, Truffle, *Tuber*

## Abstract

**Background:**

There are estimated 180–220 species of *Tuber* described in the world, but the diversity of the genus in Taiwan is poorly known, with only two species recorded, i.e., *Tuber formosanum* and *T. furfuraceum.* During our survey of hypogenous fungi in Taiwan, a whitish truffle belongs to Puberulum clade was collected from roots of *Keteleeria fortunei* var. *cyclolepis* in central Taiwan and appeared to differ from the two recorded species.

**Results:**

The whitish truffle is herein described as a new species *Tuber elevatireticulatum*, which is distinguished from closely resembled Asian whitish truffles species like *Tuber thailandicum*, *T. panzhihuanense*, *T. latisporum* and *T. sinopuberulum* by the association with *Keteleeria* host, small light brown ascocarps with a dark brown gleba, dark brownish and elliptical ascospores ornamented with a prominently raised alveolate reticulum. Molecular phylogenetic analyses of both ITS and LSU loci clearly supports *T. elevatireticulatum* as a new species without any significant incongruence.

**Conclusions:**

The whitish truffle is herein described as a new species *T. elevatireticulatum* based on the evidence from morphology and DNA sequences. *T. elevatireticulatum* is the first scientific record of whitish truffle in Taiwan.

**Electronic supplementary material:**

The online version of this article (10.1186/s40529-018-0241-y) contains supplementary material, which is available to authorized users.

## Background

True truffles, belonging to the genus *Tuber* (Tuberaceae, Pezizales, Pezizomycetes), produce hypogeous ascocarps, which are formed in soil or sometimes within layers of leaf litter. They have lost the ability to actively discharge ascospores (Bonito and Smith 2016). They are symbiotic fungi that develop association with fine roots of specific host trees (*T. oregonense* Trappe, Bonito and P. Rawl. with Douglas fir) or broad host ranges (*T. aestivum* (Wulfen:Fr.) Spreng. with some plant species in Betulaceae, Corylaceae, Fagaceae, Tiliaceae, Pinaceae and Cistaceae) (Hall et al. 2007). The unique aroma makes some species greatly sought after as high-end culinary ingredients throughout the world, especially in Europe (Hall et al. 2007). The scarcity and irreplaceably scent of French Périgord black truffle (*T. melanosporum* Vittad.) and Italian Alba white truffle (*T. magnatum* Pico.) render them among the most famous and demanding truffles in the world (Hall et al. 2007; Bonito et al. 2010a).

*Index Fungorum* (http://www.indexfungorum.org/names/Names.asp) lists out three hundred and five *Tuber* names, however, many of them required clarification (Suwannarach et al. 2015; Kinoshita et al. 2016). Bonito et al. (2013) reassessed the published names and estimated 180–220 accepted species in the genus, was subdivided into 11 major clades according to their phylogenetic relationships. Puberulum clade, Maculatum clade and closely related lineage Gibbosum clade were phylogenetically grouped with as Puberulum Group and members of this group commonly called “whitish truffle” in order to distinguish them from Italian white truffle (*T. magnatum* in Aestivum clade) (Bonito et al. 2010a; Lancellotti et al. 2016). Researches in *Tuber* have a long history and are well-documented in Europe and North America. However, research in Asia are still scarce despite the estimated high diversity (Bonito et al. 2010a; Kinoshita et al. 2011). Hypogeous fungi in Taiwan are poorly documented, with only *T. formosanum* Hu (invalidly described in 1992 due to the lack of designated holotype and later re-typification in 2013) and *T. furfuraceum* Hu and Wang reported previously. Both species form symbiotic association with roots of *Quercus glauca* (Thunb. *ex* Murray) Oerst. in the family of Fagaceae (Hu 1992; Hu and Wang 2005; Qiao et al. 2013). A whitish truffle was mentioned in Hu (1987) but lacks a formal description.

During our survey of hypogenous fungi in Taiwan, a whitish truffle was found under *Keteleeria fortunei* var. *cyclolepis* (Flous) Silba, in Sitou Tract, Nantou County of central Taiwan. It resembles several known Asian whitish truffles in the Puberulum Clade, such as *T. thailandicum* Suwannarach et al. (2015), *T. panzhihuanense* Deng et al. (2013), *T. latisporum* Chen and Liu (2007), *T. pseudosphaerosporum* Fan and Yue (2013), and *T. alboumbilicum* Wang and Li (Li et al. 2014), but differs from species in the Puberulum clade by the only species associated with *Keteleeria* host, small light brown ascocarps with hyphae-like hairs arised, dark brownish and elliptical ascospores ornamented with a prominently raised alveolate reticulum.

## Methods

### Sample collection

Ascocarps were collected with three-pronged garden cultivators, wrapped with tissue paper and kept in separate plastic zipper bags until further morphological and molecular analyses in laboratory. Ascocarps were weighted freshly within 24 h, and the pH value of adjacent soil were measured by JENCO 6010M pH meter following protocol of the manufacturer.

### Morphological analysis

Ascocarps were cleaned with dry toothbrush, and then cut into halves for observing gleba color or color change under air exposure. Sections of fresh tissue were made with a razor blade by hand, then mounted in 0.1% (w/v) cotton blue in lacto-phenol for describing morphological characteristics by a Leica DMLB light microscope. Ascospore dimensions, with the ornamentation excluded, were based on at least 100 randomly selected ascospores. The range of ascospore length to width ratio (Q), average Q with ± standard deviation (**Q**) was calculated, and number of meshes across the ascospore width was measured.

For scanning electron microscopy (SEM), ascospores from dried gleba were mounted onto SEM stubs with carbon double-sided tape (Nisshin EM CO., Ltd, Tokyo), coated with gold–palladium, then examined and photographed with a tabletop HITACHI TM3000 SEM. Holotype was deposited at Herbarium of Taiwan Forestry Research Institute, Taipei, Taiwan (Index Herbarium: TAIF).

### Molecular analysis

#### DNA extraction

Approximately 9–14 mg of gleba tissue of fresh ascocarps were ground by plastic pestle with 800 µl of Lysis Buffer (Taiwan Advanced Nanotech Inc.; containing Guanidine salt, Tris buffer and surfactants) in 1.5 ml centrifuge tube for DNA extraction. DNA was then extracted using the TANBead^Ⓡ^ fungal Nucleic Acid Extraction Kit and TANBead^Ⓡ^ Nucleic Acid Extractor (Taiwan Advanced Nanotech Inc.) following protocol of the manufacturer.

#### Polymerase chain reaction (PCR) amplification and sequencing

Two nuclear ribosomal DNA loci were used for amplifying and sequencing, including the internal transcribed spacer (ITS) with forward primer ITS5 (5′-GGAAGTAAAAGTCGTAACAAGG-3′) was paired with reverse primer ITS4 (5′-TCCTCCGCTTATTGATATGC-3′) (White et al. 1990); and ribosomal large subunit (LSU) with forward primer LR0R (5′-ACCCGCTGAACTTAAGC-3′) (Rehner and Samuels 1994) was paired with reverse primer LR5 (5′-TCCTGAGGGAAACTTCG-3′) (Vilgalys and Hester 1990). PCR was performed in 25 µl reactions containing 2.5 µl DNA template, 1 µl primer each, 8 µl ddH_2_0 and 12.5 µl 2× *Taq* Master Mix (including 20 mM KCl, 4 mM MgSO_4_·7H_2_O, 40 mM Tris–HCl with pH 8.8, 0.2% Triton X-100, 20 mM (NH_4_)_2_SO_4_, 0.2 mg/ml BSA, 0.4 mM dNTP mix, 100 U/ml *Taq* DNA Polymerase and stabilizers) (Genomics Bioscience and Technology CO., Ltd.). PCR for ITS/LSU were run as an initial denaturation at 94/95 °C for 3/2 min, then at 94/95 °C for 30 s, annealing at 56/50 °C for 30 s, extension at 72 °C for 30 s/1 min by 30 cycles and a final extension at 72 °C for 5/10 min on a multigene thermal cycler (Labnet International, Inc.). PCR products were checked on agarose gel containing 1.4% agarose and 0.5× Tris–acetate-EDTA (TAE) and stained with 5 µl/100 ml Healthview™ nucleic acid stain under UV light by multilmage™ light cabinet (Alphalmager 2200). The PCR products were sent to Seeing Bioscience Co., Ltd. for purification and sequencing by Sanger Sequencing Method (ABI 3730).

#### Phylogenetic analyses

Six ITS and eight LSU sequences were obtained from ascocarps of *T. elevatireticulatum* and were submitted to GenBank with Accession Numbers MF540616–MF540621 (ITS) and LSU sequences: LC425119–LC425126 (LSU). Other whitish *Tuber* sequences were obtained from GenBank database for phylogenetic analyses (Table 1), with *Choiromyces alveolatus* as the outgroup. Sequences were aligned using MAFFT 7 (Katoh and Standley 2013) with default settings, and poorly aligned sites were identified using Gblocks 0.91b (Castresana 2000) with gaps allowed in conserved blocks and with all other parameters left as default values. Ambiguous sites were excluded from phylogenetic analyses. Maximum likelihood (ML) analyses were conducted with MEGA 6.0 (Tamura et al. 2013) using K2P model. Bootstrap analyses were conducted with 1000 replications (Felsenstein 1985). Bayesian phylogenetic analyses were conducted with MrBayes 3.2.6 (Ronquist et al. 2012), for evaluating the effect of different phylogenetic approach. K2P model was used and MCMC chains were run for 1,000,000 generations, sampling every 100th tree. Among these, the first 20% trees were discarded as burn-in phase and the remaining trees were used to calculate Bayesian posterior probabilities. The consensus tree was viewed with FigTree 1.4.3 (Rambaut 2014).

**Table 1:** Details of the whitish *Tuber* ITS sequences used in phylogenetic study

Taxa	Voucher no.	Origin	GenBank Accession no.		References
			ITS	LSU	
*Choiromyces alveolatus*	MES97	USA	HM485332		Bonito et al. (2010a)
*Choiromyces alveolatus*	HS2886	USA	HM485333		Bonito et al. (2010a)
*Choiromyces alveolatus*	p688L	USA		EU669426	Unpublished
*Choiromyces alveolatus*	MES97	USA		JQ925660	Bonito et al. (2013)
*T. alboumbilicum*	YAAS L2324^a^	China	KJ742702		Li et al. (2014)
*T. bellisporum*	JT7270	USA	FJ809856	FJ809827	Bonito et al. (2010b)
*T. bellisporum*	JT6060	USA	FJ809857	FJ809828	Bonito et al. (2010b)
*T. borchii*	GB45	Italy	HM485344		Bonito et al. (2010a)
*T. borchii*	CMI-UNIBO 3405	Italy	FJ554521		Bonuso et al. (2010)
*T. borchii*	Tar042	Italy	KT165326		Belfiori et al. (2016)
*T. borchii*	AH39139	Spain		JN392291	Alvarado et al. (2012)
*T. borchii*	GB32	Italy		FJ809852	Bonito et al. (2010b)
*T. californicum*	JT22590	USA	HM485351		Bonito et al. (2010a)
*T. californicum*	src880	USA	HM485350		Bonito et al. (2010a)
*T. californicum*	RPC-9	USA		AF156927	Taylor and Bruns (1999)
*T. castellanoi*	JT19924	USA	FJ809859	FJ809830	Bonito et al. (2010b)
*T. castellanoi*	JT28069	USA	FJ809860	FJ809831	Bonito et al. (2010b)
*T. dryophilum*		Italy	AF003917		Unpublished
*T. dryophilum*	GB37	Italy	HM485354	JQ925688	Bonito et al. (2013)
*T. dryophilum*	GB35	Italy		JQ925687	Bonito et al. (2013)
***T. elevatireticulatum***^*b*^	**XTAM1**	**Taiwan**	**MF540616**	**LC425119**	**This study**
***T. elevatireticulatum***	**XTAM2**	**Taiwan**	**MF540617**	**LC425120**	**This study**
***T. elevatireticulatum***	**XTAM3**^a^	**Taiwan**	**MF540618**	**LC425121**	**This study**
***T. elevatireticulatum***	**XTAM4**	**Taiwan**	**MF540619**	**LC425122**	**This study**
***T. elevatireticulatum***	**XTAM5**	**Taiwan**	**MF540620**		**This study**
***T. elevatireticulatum***	**XTAM7**	**Taiwan**	**MF540621**	**LC425123**	**This study**
***T. elevatireticulatum***	**XTBX1**	**Taiwan**		**LC425124**	**This study**
***T. elevatireticulatum***	**XTBX4**	**Taiwan**		**LC425125**	**This study**
***T. elevatireticulatum***	**XTBX5**	**Taiwan**		**LC425126**	**This study**
*T. flavidosporum*	K213^a^	Japan	AB553446	AB553520	Kinoshita et al. (2016)
*T. gibbosum*	SPCP_B2a	Canada	KP972062		Berch and Bonito (2016)
*T. gibbosum*	JT6555	USA		FJ809833	Bonito et al. (2010a)
*T. gibbosum*	JT19424	USA	HM485362	FJ809834	Bonito et al. (2010a)
*T. huizeanum*	BJTC FAN186^a^	China	JQ910651	NG_059991	Fan et al. (2013a)
*T. japonicum*	N88^a^	Japan	AB553444		Kinoshita et al. (2016)
*T. japonicum*	K228	Japan		AB553519	Kinoshita et al. (2016)
*T. latisporum*	HKAS 44315^a^	China	DQ898183		Chen and Liu (2007)
*T. latisporum*	BJTC FAN126	China		KP276204	Fan et al. (2016a)
*T. lijiangense*	BJTC FAN307	China	KP276188	KP276203	Fan et al. (2016a)
*T. liui*	HKAS 48269	China	DQ898182		Chen and Liu (2007)
*T. liyuanum*	BJTC FAN162^a^	China	JQ771191		Fan and Cao (2013)
*T. liyuanum*	BJTC FAN162^a^	China		KT067698	Fan et al. (2016b)
*T. maculatum*	M4TM	Poland	KJ524530		Unpublished
*T. maculatum*	Mac1	Italy	AF106889		Unpublished
*T. maculatum*	ZB2656	Hungary		JF261366	Unpublished
*T. microsphaerosporum*	BJTCFan152^a^	China	KF805726		Fan and Yue (2013)
*T. microverrucosum*	BJTC FAN142^a^	China	JN870099		Fan et al. (2011)
*T. microverrucosum*	BJTC FAN142^a^	China		KT067696	Fan et al. (2016b)
*T. oligospermum*	AH39338	France	JN392266	JN392319	Alvarado et al. (2012)
*T. oligospermum*	AH37867	Italy	JN392259	JN392322	Alvarado et al. (2012)
*T. oregonense*	SPCP_B26	Canada	KP972064		Berch and Bonito (2016)
*T. oregonense*	DUKE GB284^a^	USA	FJ809874		Bonito et al. (2010b)
*T. oregonense*	JT27945	USA		FJ809836	Bonito et al. (2010b)
*T. oregonense*	JT8767	USA		FJ809837	Bonito et al. (2010b)
*T. panzhihuanense*	DXJ267^a^	China	JQ978648		Deng et al. (2013)
*T. panzhihuanense*	HKAS:95329			KY174963	Unpublished
*T. panzhihuanense*	HKAS:95328			KY174962	Unpublished
*T. pseudomagnatum*	BJTC FAN163^a^	China	JQ771192		Fan and Cao (2013)
*T. pseudomagnatum*	BJTC FAN163^a^	China		KP276192	Fan et al. (2016b)
*T. pseudosphaerosporum*	BJTCFan250^a^	China	KF744063		Fan and Yue (2013)
*T. pseudosphaerosporum*	BJTCFan250^a^	China		KP276194	Fan et al. (2016a)
*T. puberulum*		Serbia	FM205642		Marjanovic et al. (2010)
*T. puberulum*	ZB436	Hungary		JF261369	Unpublished
*T. shearii*	OSC51052	USA	HM485389		Bonito et al. (2010a)
*T. shearii*	OSC51052	USA		JF419280	Guevara et al. (2013)
*T. shearii*	JT12498	USA	GQ221450		Unpublished
*T. sinopuberulum*	BJTC FAN157^a^	China	JQ690073	JQ690070	Fan et al. (2013b)
*T. sinosphaerosporum*	BJTC FAN135^a^	China	JX092086		Fan et al. (2013c)
*T. sinosphaerosporum*	BJTC FAN135^a^	China		KP276195	Fan et al. (2016a)
*T. sphaerospermum*	AH37798	Morocco	JN392245	JN392304	Alvarado et al. (2012)
*T. sphaerospermum*	AH39197	Spain	JN392242	JN392307	Alvarado et al. (2012)
*T. thailandicum*	CMU-MTUF1^a^	Thailand	KP196328	KP196333	Suwannarach et al. (2015)
*T. thailandicum*	CMU-MTUF2	Thailand	KP196329	KP196334	Suwannarach et al. (2015)
*T. turmericum*	BJTC FAN473^a^	China	KT758837		Fan et al. (2015)
*T. vesicoperidium*	BJTC FAN155^a^	China	JQ690071	JQ690068	Fan et al. (2013b)
*T. xanthomonosporum*	YAAS L3185^a^	China	KJ162154		Qing et al. (2015)
*T. zhongdianense*	wang0299^a^	China	DQ898187		Chen and Liu (2007)
*T. zhongdianense*	BJTC FAN176	China		KP276201	Fan et al. (2016a)

^a^Holotype

^b^New species described in this study are bold as indication

## Results

### Taxonomy

**Fig. 1:**
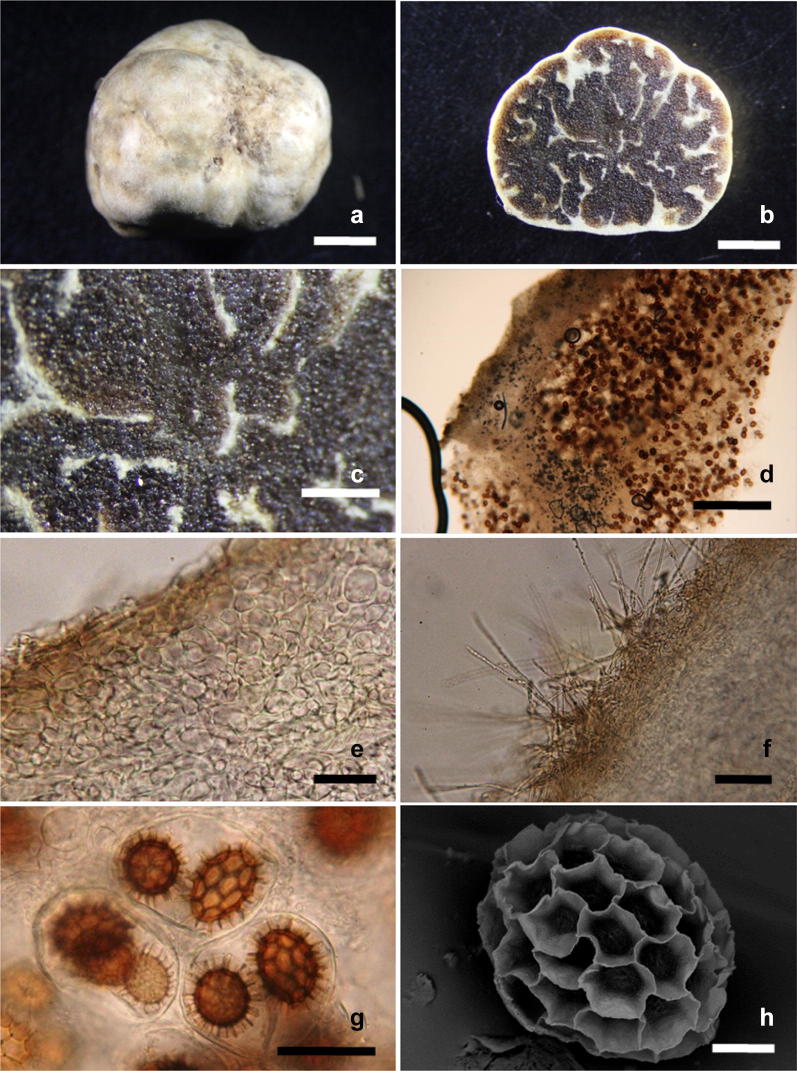
*Tuber elevatireticulatum*. **a** Mature ascocarp. **b**, **c** Cross section of ascocarp showing a dark brown gleba with narrow, light brown veins. **d** Section of peridium and gleba. **e** Pseudoparenchymatous tissue of peridium. **f** Hyphae-like hairs arising from outermost cells. **g** Ascospores. **h** Scanning electron micrograph of an ascospore. *Bars:* **a**, **b** 3.5 mm; **c** 1.5 mm; **d** 500 µm; **e**–**g** 50 µm; **h** 10 µm

***Tuber elevatireticulatum*** **K.F. Wong and H.T. Li, sp. nov. Fig.** 1

MycoBank no.: MB824068.

Etymology: Referring to the prominently elevated reticulum on the ascospores.

*Ascocarp* hypogeous, scattered, solitary, subglobose or irregular, 12–19 mm long × 10–15 mm wide, 0.32–1.7 g in fresh weight, solid, smooth on the surface, whitish to pale yellowish when fresh, becoming light brown at maturity. *Peridium* two-layered; inner layer 85–425 μm thick, hyaline, composed of intricately interwoven hyphae; outer layer 75–110 μm thick, light brownish, pseudoparenchymatous, composed of globose, subglobose, rod-shaped or angular cells, 5–25 μm diam. Hyphae-like hairs arise from outermost cells, hyaline, septate, tapering towards the ends, acute or round at the apex, 50–275 × 1.25–3.75 μm. *Gleba* translucent or light-brown, marbled with narrow, white veins when young, becoming dark brown, marbled with narrow, light brown veins at maturity. *Asci* 1-3(-4)-ascospored, globose, subglobose, ovoid to ellipsoid, 47.5–88 × 37.5–75 µm, hyaline, with a wall 2.5 µm thick. *Ascospores* broadly ellipsoid to ellipsoid, rarely subglobose and globose, with mature ascospore ratio ranging 0.2–53% (n = 1000), yellowish brown to dark brown, with a wall 2.5–5 µm thick, 32.5–50 × 20–32.5 µm from 1-ascospored asci, 20–48 × 20–32.5 µm from 2-ascospored asci, 20–40 × 20–27.5 µm from 3-ascospored asci, 22.5–35 × 17.5–25 µm from 4-ascospored asci (Q = 1.0–1.75, **Q **= 1.30 ± 0.19), ornamented with irregular reticulations 2.5–7.5 µm high, with meshes varying in size, mostly 3-4(-5) across the ascospore width.

Specimens examined: TAIWAN, Nantou County, Sitou Tract, associated with roots of *K. fortunei* var. *cyclolepis*, 1 Jun 2017, collected by C.-L. Lin, K.-F. Wong, H.-T. Li and F.-Y. Lin, XTAM3 (holotype), ITS sequences: MF540616–MF540621; LSU sequences: LC425119–LC425126.

Notes: *Tuber elevatireticulatum* grows in montane area of central Taiwan with elevation of 1150 m. It is associated with a cluster of *K. fortunei* var. *cyclolepis* in a mixed coniferous plantation, at least 4 m apart from the nearest *Cryptomeria japonica* (L. f.) D. Don, *Chamaecyparis formosensis* Matsum. and a few *Pinus* species which all have no record of association with *Tuber* species. Ascocarps are mostly scattered and distributed in solitary in loamy soil with pH ranging from 5 to 6. Ascocarps are usually found within 0–2 m from tree trunks, starting to develop in March and maturing in June. Odor is pleasant, mild, peculiar but superb, rarely becoming unpleasant with ageing. The temperature during the ascocarp formation is 20–25 °C.

### Phylogenetic analyses

**Fig. 2:**
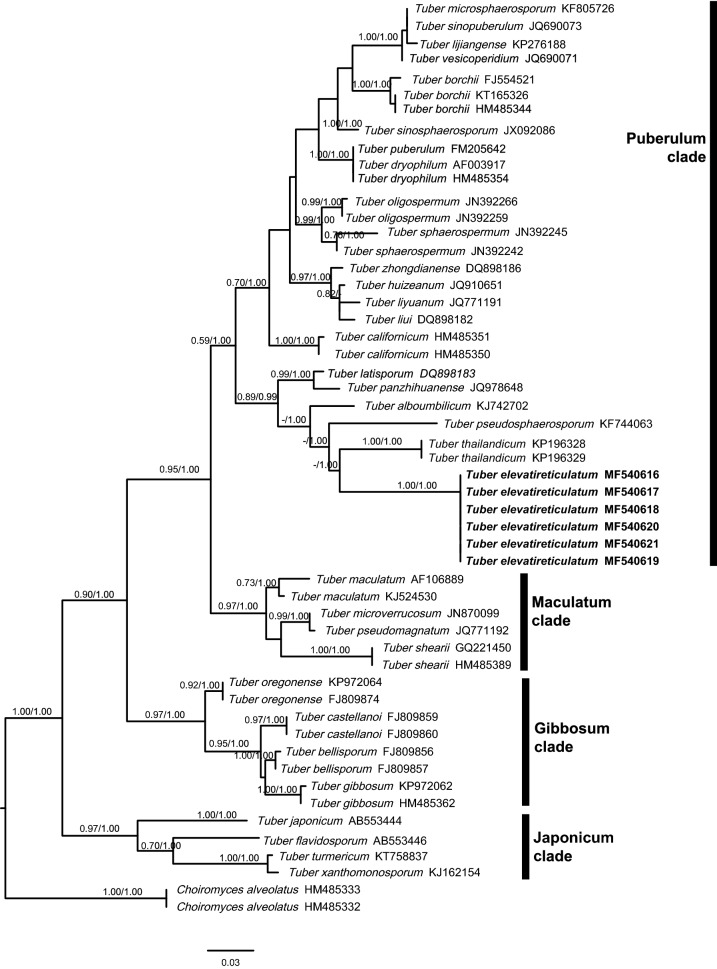
Phylogenetic tree of *Tuber elevatireticulatum* and related whitish truffles based on the ITS-rDNA sequences. *Choiromyces alveolatus* was used as the outgroup taxa. Numbers identify the bootstrap values and Bayesian posterior probabilities are indicated near branches as BS/PP. Values of BS and PP below 50% are not indicated. The sequences of new species described in this study are bold as indication

**Fig. 3:**
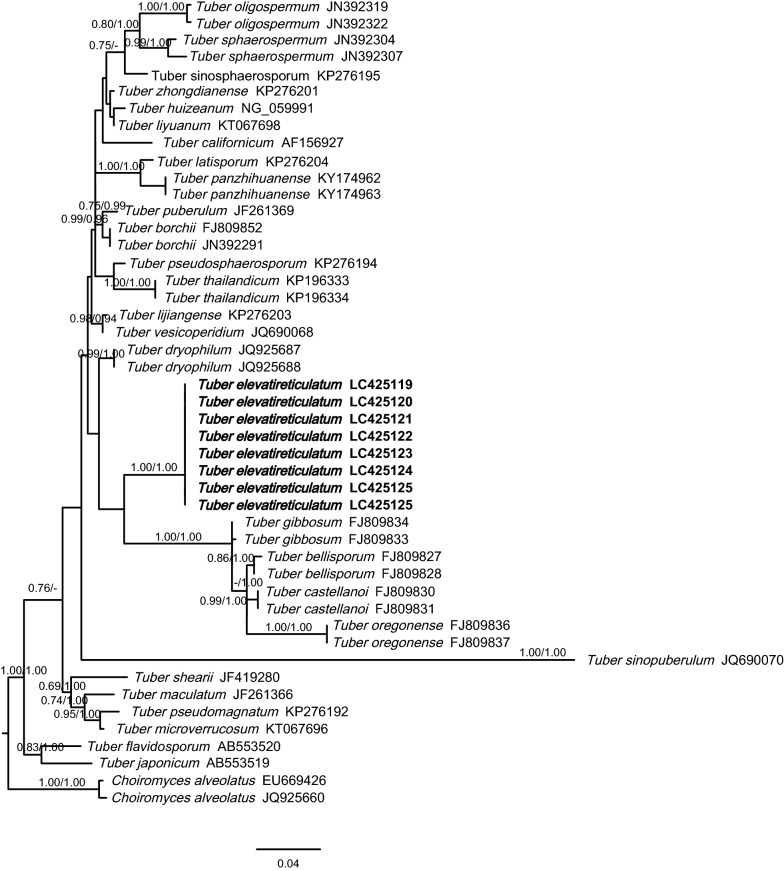
Phylogenetic tree of *Tuber elevatireticulatum* and related whitish truffles based on the LSU-rDNA sequences. *Choiromyces alveolatus* was used as the outgroup taxa. Numbers identify the bootstrap values and Bayesian posterior probabilities are indicated near branches as BS/PP. Values of BS and PP below 50% are not indicated. The sequences of new species described in this study are bold as indication

The ITS matrix consisted of 52 sequences and 1661 aligned bases, of which 1198 bp were identified as poorly aligned and were excluded by Gblocks. The resultant ITS alignment was 463 bp. The LSU matrix consisted of 47 sequences and 1519 aligned bases, of which poorly aligned and were excluded by Gblocks and the resultant LSU alignment was 580 bp. As Maximum likelihood and Bayesian analyses yielded similar tree topologies of ITS region, thus the only tree generated form ML analysis is shown in Fig. 2. The ML and Bayesian analyses of LSU region is similar in general, due to the limited availability of sequences in database, the tree inferred form ML analysis is presented in Fig. 3, separate trees are presented as Additional files 1, 2.

There has no significant incongruence among ITS and LSU region of ribosomal DNA. *Tuber elevatireticulatum* is clearly different from other whitish truffles and formed a monophyletic clade with strong bootstrap (BS) and posterior probability (PP) values (1.00/1.00). Based on the ITS analysis, *T. elevatireticulatum* was placed clearly in the Puberulum clade, within which it formed a subclade with five Asian species, including *T. thailandicum*, *T. pseudosphaerosporum*, *T. alboumbilicum*, *T. latisporum*, and *T. panzhihuanense*, with strong branching supports by BS (0.89) and PP (0.99) value. Also included in the Puberulum clade were *T. borchii*, *T. dryophilum, T. oligospermum* and *T. sphaerospermum* from Europe; *T. microsphaerosporum, T. sinopuberulum*, *T. vesicoperidium, T. lijiangense*, *T. sinosphaerosporum, T. zhongdianense, T. huizeanum*, *T. liui* and *T. liyuanum* from China; and *T. californicum* from the USA. These whitish truffle species formed a subclade within the Puberulum clade with strong PP value of 1.00 and was sister to the one where *T*. *elevatireticulatum* was placed. The groupings of whitish truffles were similar from those in Kinoshita et al. (2011), Suwannarach et al. (2015) and Bonito and Smith (2016).

## Discussion

*Tuber elevatireticulatum* is distinguished from other whitish truffle species by the only species associated with *Keteleeria* host, its small light brown ascocarps with a dark brown gleba and brown, ellipsoid ascospores with a prominent raised alveolate reticulum. Phylogenetic analyses clearly placed *T. elevatireticulatum* among other whitish truffle species in the Puberulum clade as a distinct taxon. Morphologically, truffles belonging to the Puberulum clade tend to have small and light-colored ascocarps, globose to subglobose ascospores with an alveolate-reticulate ornamentation (Bonito and Smith 2016). However, ascospores of *T. elevatireticulatum* are mostly ellipsoid, resembling those of the species in the Maculatum clade.

*Tuber elevatireticulatum* clustered in a subclade of the *Puberulum* group with several Asian whitish truffle species, including *T*. *thailandicum*, *T*. *pseudosphaerosporum*, *T. alboumbilicum*, *T. panzhihuanense*, and *T. latisporum* (Fig. 2). *Tuber elevatireticulatum* is similar to *T. thailandicum* in having a dark brown gleba at maturity, hyphae-like hairs, and the size of alveolae of the reticulum. However, *T. thailandicum* differs by having a larger ascocarp size (> 2 cm in diam.), a thinner peridium (150–225 µm), shorter hyphae-like hairs (20–63.5 µm), subglobose ascospores with a smaller **Q** value (1.09 ± 0.08), and larger ascospores in one-ascospored asci (40–65 × 40–62 µm) (Suwannarach et al. 2015). In addition, *T. thailandicum* is associated with roots of *Betula*, whereas *T. elevatireticulatum* is with *Keteleeria* roots, a host previously unknown to *Tuber* species. *Tuber elevatireticulatum* resembles *T. pseudosphaerosporum* in having light-colored ascocarps with a smooth surface and the same numbers of ascospores in asci but differs from the latter by a smaller ascocarp size, well-developed hyphae-like hairs, larger ellipsoid ascospores, a lower reticulum, and occurrence in a different season (Fan and Yue 2013). *Tuber alboumbilicum* is different from *T. elevatireticulatum* by a smaller ascocarp size (< 1 cm), a thinner peridium, and globose ascospores. *Tuber panzhihuanense* is distinct from *T. elevatireticulatum* by a dark grey to blackish gleba (Deng et al. 2013). *Tuber latisporum* is different from *T. elevatireticulatum* by reddish brown ascocarps, a blackish gleba and larger ascospores (62–93 × 41–74 µm) (Chen and Liu 2007). Beyond this subclade, *Tuber sinopuberulum* resembles *T. elevatireticulatum* in having light brown ascocarps with a smooth surface but differs from it in lacking hyphae-like hairs arising from the peridium, a light brown to brown gleba color, and globose ascospores (Fan et al. 2012).

Truffles in general favor dry, alkaline and calcareous soil (Hall et al. 2007), but *T. elevatireticulatum* was found in an area with a subtropical humid climate, slightly acidic soil of pH 5–6, and relatively high annual rainfall. This phenomenon has also been observed in Asia like Japan (Kinoshita et al. 2011) and Thailand (Suwannarach et al. 2015).

## Additional files

**Additional file 1.**  Phylogenetic tree of *Tuber elevatireticulatum* and related whitish truffles based on the ITS-rDNA sequences by Bayesian phylogenetic analyses

**Additional file 2.** Phylogenetic tree of *Tuber elevatireticulatum* and related whitish truffles based on the LSU-rDNA sequences by Bayesian phylogenetic analyses.
