# Supplementary figures and images for: Tuber elevatireticulatum sp. nov., a new species of whitish truffle from Taiwan
Source: Bot Stud. 2018 Oct 29;59:25. doi: 10.1186/s40529-018-0241-y (PMC6206313; doi:10.1186/s40529-018-0241-y)

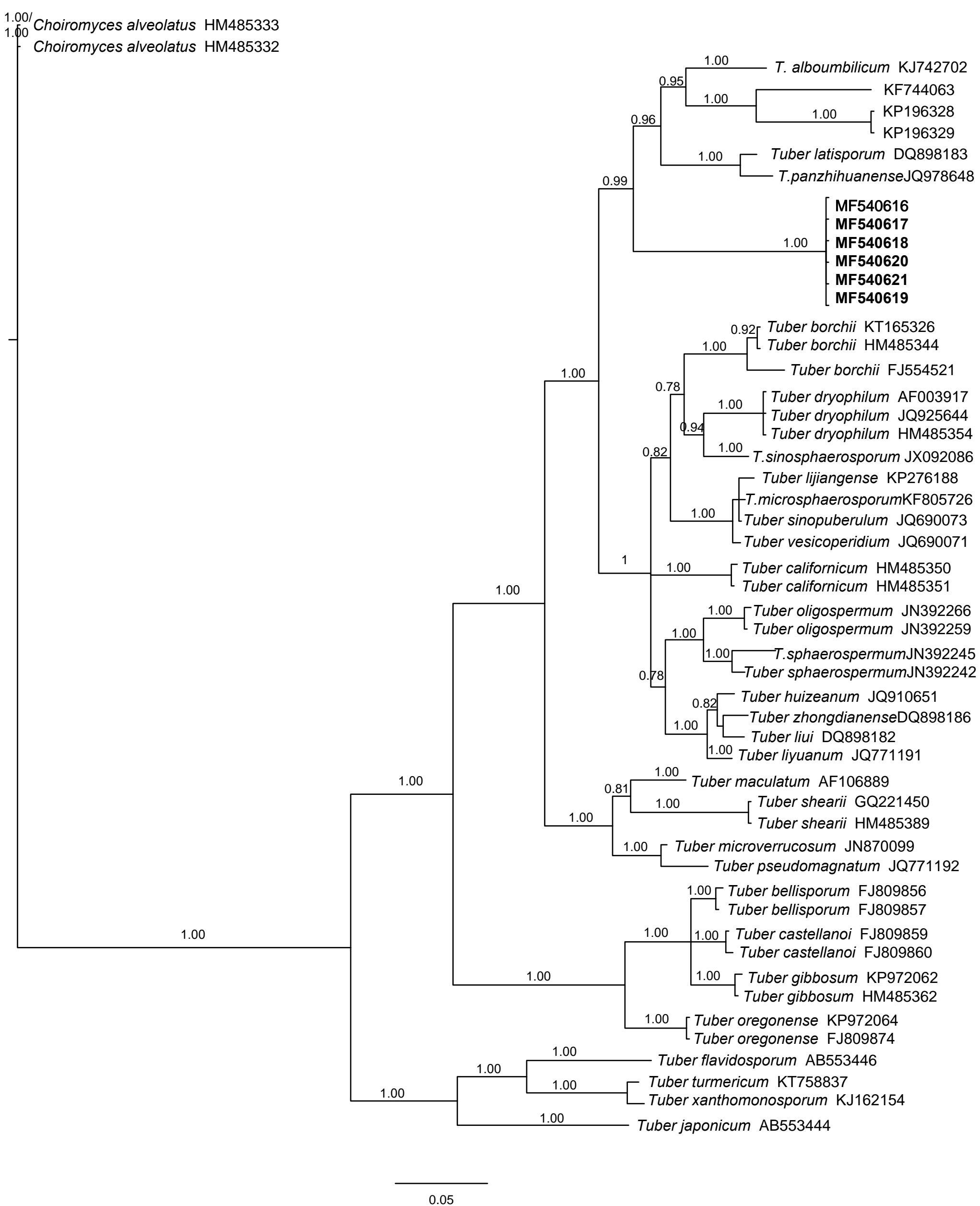

Supplement: Supplementary file 1 — Additional file 1. Phylogenetic tree of Tuber elevatireticulatum and related whitish truffles based on the ITS-rDNA sequences by Bayesian phylogenetic analyses [file 40529_2018_241_MOESM1_ESM.pdf]

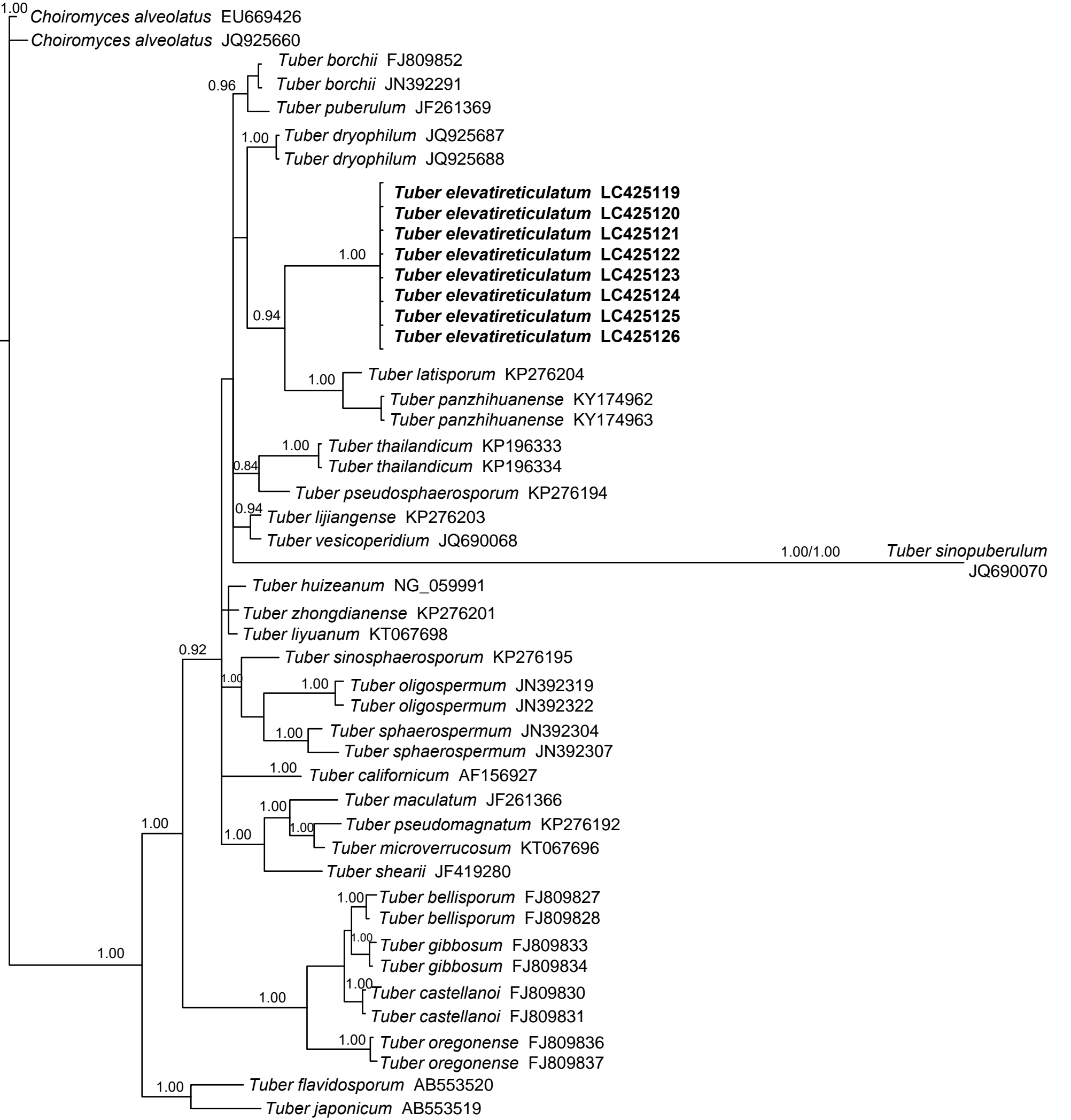

0.04

Supplement: Supplementary file 2 — Additional file 2. Phylogenetic tree of Tuber elevatireticulatum and related whitish truffles based on the LSU-rDNA sequences by Bayesian phylogenetic analyses. [file 40529_2018_241_MOESM2_ESM.pdf]
